# Rational design of gold nanocarrier for the delivery of JAG-1 peptide

**DOI:** 10.1186/s12951-015-0100-x

**Published:** 2015-06-16

**Authors:** Cristian T Matea, Teodora Mocan, Flaviu Tabaran, Cornel Iancu, Lucian-Constantin Mocan

**Affiliations:** 3rd Surgery Clinic, Department of Nanomedicine, “Iuliu Hatieganu” University of Medicine and Pharmacy, Cluj-Napoca, Romania; Department of Physiology, “Iuliu Hatieganu” University of Medicine and Pharmacy, Cluj-Napoca, Romania; Department of Pathology, Faculty of Veterinary Medicine, University of Agricultural Sciences and Veterinary Medicine, Cluj-Napoca, Romania

**Keywords:** Gold nanoparticles, JAG-1 peptide, PEG functionalization

## Abstract

**Background:**

Unique properties exhibited by nanoparticles makes them great candidates for applications in physics, chemistry, biology, material science and medicine. The biological applications of water-soluble gold nanoparticles range from contrast agents, delivery vehicles to therapeutics. Notch signaling is a complex network that orchestrates cell fate decisions, which involves proliferation, migration, differentiation and cell death in organisms ranging from insects to humans. Studies have showed that a correct orientation of the Jag-1 signalling protein on the substrates proves to be of great importance when promoting Jagged-1 Notch interactions, also the availability of the ligands, super cedes the importance of their concentration.

**Results:**

The aim of the present study was to synthetize a Jag-1 functionalized nanocarrier, which would promote an efficient interaction between the Jag-1 peptide and the Notch receptor. To this end, two routes for gold nanoparticle-peptide assembly were investigated, and the synthetized bio-nanostructures were characterized and compared by means of UV–Vis, FT-IR, DLS and AFM techniques.

**Conclusions:**

We have obtained a stable, monodisperse, hetero-functionalized GNP-PEG-JAG-1 bio-nanostructure for Notch pathway activation applications.

**Electronic supplementary material:**

The online version of this article (doi:10.1186/s12951-015-0100-x) contains supplementary material, which is available to authorized users.

## Background

Notch signaling is a complex network that orchestrates cell fate decisions, which involves proliferation, migration, differentiation and cell death in organisms ranging from insects to humans [1, 2]. Ligands such as Jagged-1,-2 and Delta-1,-3 and -4 have been found to activate the mammalian Notch transmembrane receptor (Notch-1 to -4) [1, 3, 4]. Kertesz et al. showed that Jagged-Fc adsorbed on the surface of particles enhances the proliferation of ex vivo hematopoietic stem cells [5]. Another study conducted by Nickollof et al. demonstrated that a 17 amino acid peptide dubbed Jag-1 mimics the function of full-length Jagged-1 protein and can be used as an agonist in order to activate the Notch pathway [2]. Studies have showed that a correct orientation of the Jag-1 signalling protein on the substrates proves to be of great importance when promoting Jagged-1 Notch interactions, also the availability of the ligands, super cedes the importance of their concentration [1, 4].

Unique properties exhibited by nanoparticles makes them great candidates for applications in physics, chemistry, biology, material science and medicine [6]. In recent years, gold nanoparticles have gained great attention in the eyes of the medical community, firstly, due to the fact that the gold core is essentially inert and non-toxic [7]. The biological applications of water-soluble gold nanoparticles range from contrast agents, delivery vehicles to therapeutics [8]. Another key feature for these gold nanostructures is the ease of synthesis of mono-disperse nanoparticles with sizes ranging from 1 to 150 nm [7]. The most common technique used to prepare aqueous-stable gold nanoparticles for biological studies is the citrate reduction method, first featured by Turkevich et al. [9]. In this case Au^3+^ ions are reduced to metallic gold and stabilized by a citrate ligand layer through electrostatic interactions [10]. Investigations of citrate capped gold nanoparticles showed that they have a negatively charged surface and preferentially bind to thiol, amine and cyanide functional groups [11]. In order to further stabilize and modulate their physico-chemical properties, gold nanoparticles have been functionalized with a wide range of simple and complex molecules by means of their reactive functional groups [12]. For example, peptides [13], proteins [14], DNA [15], polymers [16], and dendrimers [17] were conjugated to the gold nanoparticles surface by means of non-covalent electrostatic interaction, hydrophobic interaction and covalent binding [12, 14].

Some molecular methods for incorporating peptides and oligonucleotides present themselves as relatively inefficient, limit the peptide sequence diversity because of chemistry incompatibility and can generate undesired products. In order to functionalize nanoparticles with biomolecules two types of functionalization methods are employed: homo- and heterofunctionalization. The first one refers to a straight forward approach, where only 1 biomolecule functionality is incorporated (e.g. peptide, antibody, DNA), while the latter one refers to the incorporation of 2 or more biomolecule functionalities (e.g. protein-stabilized DNA-peptide conjugate) [15, 18–21].

The aim of the present study was to synthetize a Jag-1 functionalized nanocarrier, which would promote an efficient interaction between the Jag-1 peptide and the Notch receptor. To this end, two routes for gold nanoparticle-peptide assembly were investigated, and the synthetized bio-nanostructures were characterized and compared by means of UV–Vis, FT-IR, DLS and AFM techniques.

## Methods

Choloroauric acid (HAuCl_4_, ≥99.9%), tri-sodium citrate (≥99%), disodium phosphate (Na_2_HPO_4_, ≥99%), dimethyl sulfoxide (DMSO ≥99.7%) and poly(ethylene glycol) dithiol (average M_n_ = 3,400) were purchased from Sigma-Aldrich (Darmstadt, Germany). JAG-1 peptide active fragment was purchased from StemRD (CA, USA). Reagents were used as received without further purification. All glassware used in the experiments was cleaned with aqua regia (HCl: HNO_3_, 3:1) prior to use.

JAG-1 peptide cell penetration prediction was performed with the aid of the web-based CellPPD server [22], the SVM+ motif with a threshold of 0.1 and an E-value cut-off of 10. The 3D structure of the peptide was predicted by the PEPstr [23] web-based server and the structure validated with the VADAR server [24, 25].

The MC3T3-E1 osteoblast cell line was purchased and maintained using standard cell culture protocols [European Collection of Cell Cultures (ECACC)]. When reaching the growth log-phase, test sample cells were exposed to GNP-PEG-JAG-1 by medium removal and addition GNP-PEG-JAG-1 solution in appropriate concentrations (34.5, 17.3, 8.6, 4.3, 2.1 μg mL^−1^, respectively). A volume of 400 μL/well for 4 chamber slides, or 100 μL/well for 96 well plates was used. Next, 1 h incubation (5% CO2, 37°C) was allowed for exposure. Control sample was exposed to appropriate culture medium [MEM alpha + 2 mM glutamine + 10% fetal bovine serum (FBS)].

The synthesis of gold nanoparticles was done in according to a modified Turchevich method. Briefly, 29 mg of HAuCl_4_ were dissolved in 50 mL ultrapure water under vigorous agitation. To this, a 5 mL solution of sodium citrate 50 mM was quickly added and the solution was brought to boiling. The reaction was allowed to continue for 1 h under reflux. Afterwards, the obtained ruby-red solution was cooled to room temperature and aliquots were pulled for characterization purposes.

For coupling of the JAG-1 peptide on the gold nanoparticle surface two routes were undertaken.

Route I: The pH of a 5 mL solution of citrate capped GNPs (11.5 nM) was adjusted to ~7 with the aid of a 0.1 M Na_2_HPO_4_ solution. Next, 500 μL of the JAG-1 solution 5 μM were added under continuous stirring. The reaction between the GNPs and the JAG-1 peptide was allowed to continue for 1 h under stirring at room temperature. In order to separate the functionalized GNP-JAG-1 nanocomposite from the uncoupled JAG-1 peptide the sample was subjected to a 16,000 RPM centrifugation step for 30 min. The supernatant was discarded and the obtained pellet was re-dispersed in ultrapure water by means of ultrasonication.

Route II: The pH of a 10 mL 5 nM solution of citrate capped GNPs was adjusted to ~7 with the aid of a 0.1 M Na_2_HPO_4_ solution. Next 1 mL of PEG dithiol (M_n_ = 3,400) 100 μM was added and the reaction was allowed to continue for 30 min under continuous stirring. Afterwards, 1 mL of 5 μM JAG-1 solution and 1 mL of DMSO 100 μM were added to the sample. The reaction was allowed to continue 120 min at room temperature, under continuous stirring. The conjugate was purified by centrifugation at 13,200 RPM for 20 min, and the resulting pellet was re-dispersed in ultrapure water by means of ultrasonication.

UV–Vis spectroscopy sample characterization was done on a Shimadzu UV-1800® instrument. Spectral data were collected in the 800–200 nm range, with a 0.5 nm resolution at room temperature. A 1 cm lightpath quartz cuvette was used and samples were diluted (1:10 v/v) prior to the measurements. All recorded spectras were normalized using the OriginLab software® 7.0.

Dynamic light scattering measurements were conducted on a Zetasizer—Nano S90 Malvern Instrument (Westborough, UK). A 90° scattering angle, 25 ± 0.1°C and a refractive index of 1.4 were considered for all samples. Measurements were carried out in triplicate.

Universal attenuated total reflectance Fourier-transform infrared spectroscopy (UATR-FT-IR) spectral characterization was done with the aid of a Perkin-Elmer Spectrum Two® instrument equipped with an attenuated total reflectance stage (ATR), with over 128 scans per sample and a resolution of 1 cm^−1^. Baseline corrections and spectra processing were done with the Spectrum 10 software.

Atomic force microscopy images were collected with a Workshop TT-AFM® instrument (AFMWorkshop, CA, USA), operating in vibrating mode with ACTA-SS cantilevers (AppNano, CA, USA). Samples were deposited on a mica surface by means of a spin coater (KLM® SCC). The collected images were processed with the Gwyddion® software ver. 2.36.

High contrast dark-field imaging (100×) of GNP and GNP-PEG-JAG-1 samples was done on an Olympus BX-43 microscope with a CytoViva® modification. For each sample, an amount of 15 μL was deposited on a glass slide and sealed with a cover slip.

Cell growth and viability was assessed by measuring the ability to cleave 3-[4, 5-dimethylthiazol-2-yl]-2, 5-diphenyl tetrazolium bromide (MTT). For performing the assay (Molecular Probes), cells were seeded into a 96 well plate. Briefly, following exposure, cells were incubated with the MTT solution (4 h, 37°C). Next, SDS-HCl addition was performed, followed by incubation (4 h, 37°C). Absorbance was read spectrophotometrically at 570 nm using the fluorescence modulus of a Perkin Elmer Viktor multilabel multitask plate reader. Experiments were performed in triplicate. Results were expressed as percentage calculated from non-exposed group’s absorbance. SPSS 17.0 (Chicago, Il) statistical package was used for data analysis. Kruskall Wallis and Mann–Whitney U Test were used to assess overall among-group and two-group differences respectively. A threshold of p < 0.05 was considered significant. Data was expressed as mean (std. error).

## Results

Gold nanoparticles were synthetized by employing a modified Turchevich method. This approach yields mono-disperse citrate-capped metallic gold nanostructures. The obtained GNPs showed themselves as stable in aqueous media over several weeks and were characterized by means of UV–Vis, ATR-FT-IR, and DLS techniques. The surface plasmon resonance band (SPR) for the GNP solution, as determined by UV–Vis spectroscopy, was located at 522 nm, value in agreement with other literature data [24, 26–28]. The concentration of the GNP solution was calculated to be 11.5 nM based on the extinction coefficient at 522 nm [29, 30]. Dynamic light scattering (DLS) measurements conducted on the GNPs revealed a hydrodynamic diameter of 16 ± 2 nm.

The JAGGED-1 peptide has 17 aminoacid residues (one letter code: CDDYYYGFGCNKFCRPR) of which three are cysteine residues. Data generated by CellPPD, PEPstr and VADAR peptide prediction servers showed that JAG-1 is a non-cell penetrating peptide that has a molecular weight of 2,107.37 Da, a pI = 8.22 and a predicted 3D coil type structure.

**Figure 1:**
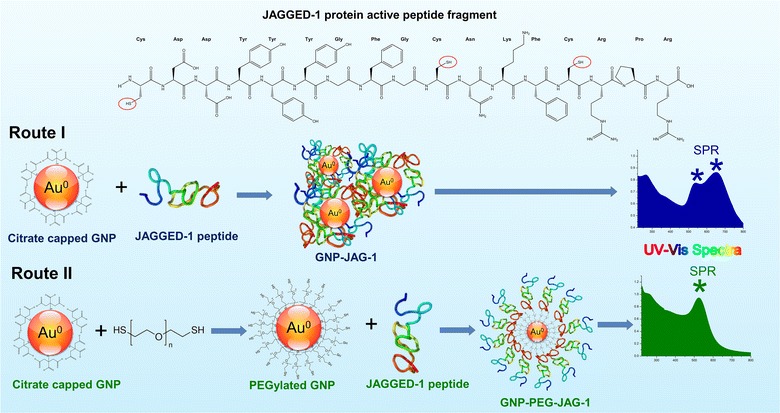
Illustration of the proposed functionalization routes for binding the JAG-1 peptide (top chemical structure) to gold nanoparticles.

In order to functionalize the obtained GNPs with the JAG-1 peptide, two routes were taken into consideration, a homo- and a heterofunctionalization approach, they are illustrated in Figure 1. Route I aimed to couple the JAG-1 peptide directly on the GNPs surface through the thiol functional groups from the cysteine residues present in the mentioned peptide. Another functionalization route (route II) investigated was the coupling of JAG-1 to a dithiol PEG spacer which, in turn, is coupled on the GNPs surface. Both nanostructures obtained, GNP-JAG-1 (from route I) and GNP-PEG-JAG-1 (from route II) were evaluated in terms of size, shape, stability and phisico-chemical properties.

**Figure 2:**
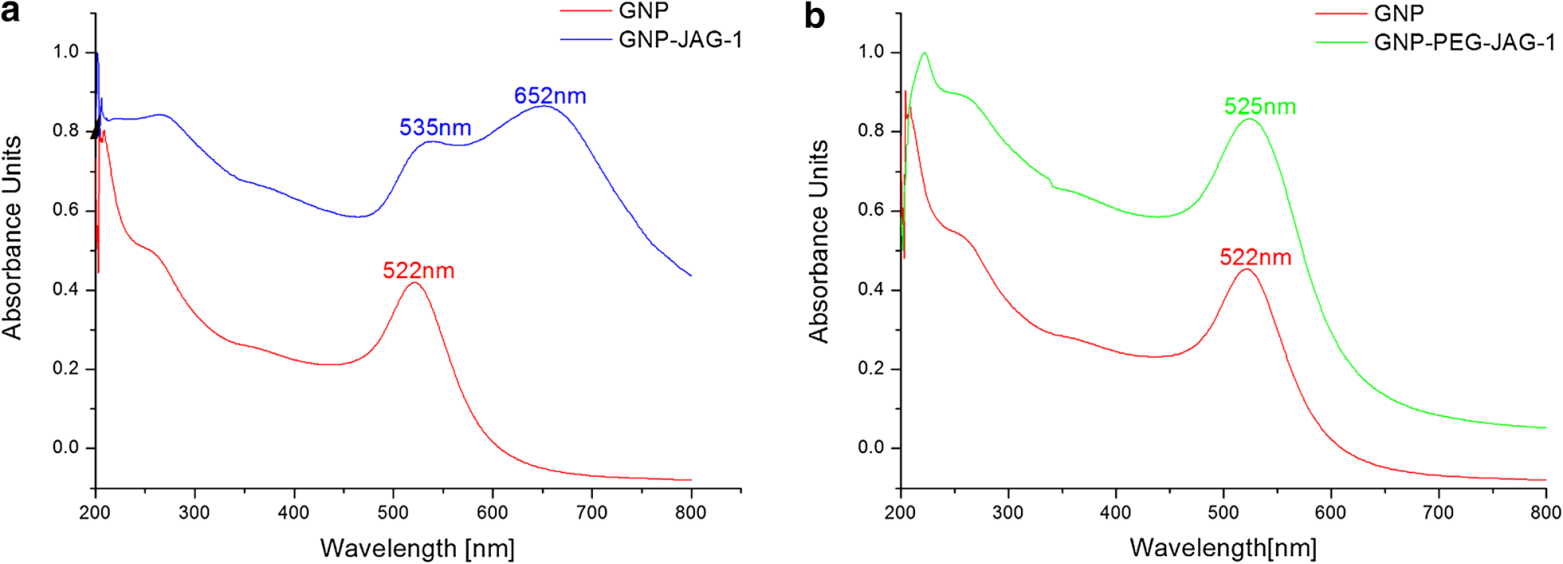
**a** UV–Vis spectra of citrate capped GNPs (*red line*) versus GNP-JAG-1 bio-nanocomposite (*blue line*) and their corresponding SPR bands. **b** UV–Vis spectra of citrate capped GNPs (*red line*) versus GNP-PEG-JAG-1 (*green line*) and their corresponding SPR bands.

UV–Vis spectroscopy measurement results are presented in Figure 2. The spectra for sample GNP-JAG-1, blue line in Figure 1a, presents two SPR bands at 535 and 652 nm. This bathochromic shift of the initial SPR band suggests that JAG-1 has been coupled on the gold nanoparticle surface, but the resulting bio-nanocomposite is polydisperse. On the other hand, the GNP-PEG-JAG-1 UV–Vis spectra registered a single SPR band (Figure 2b—green line), located at 525 nm, indicating both successful functionalization and a monodiaperse nanoparticle population.

**Figure 3:**
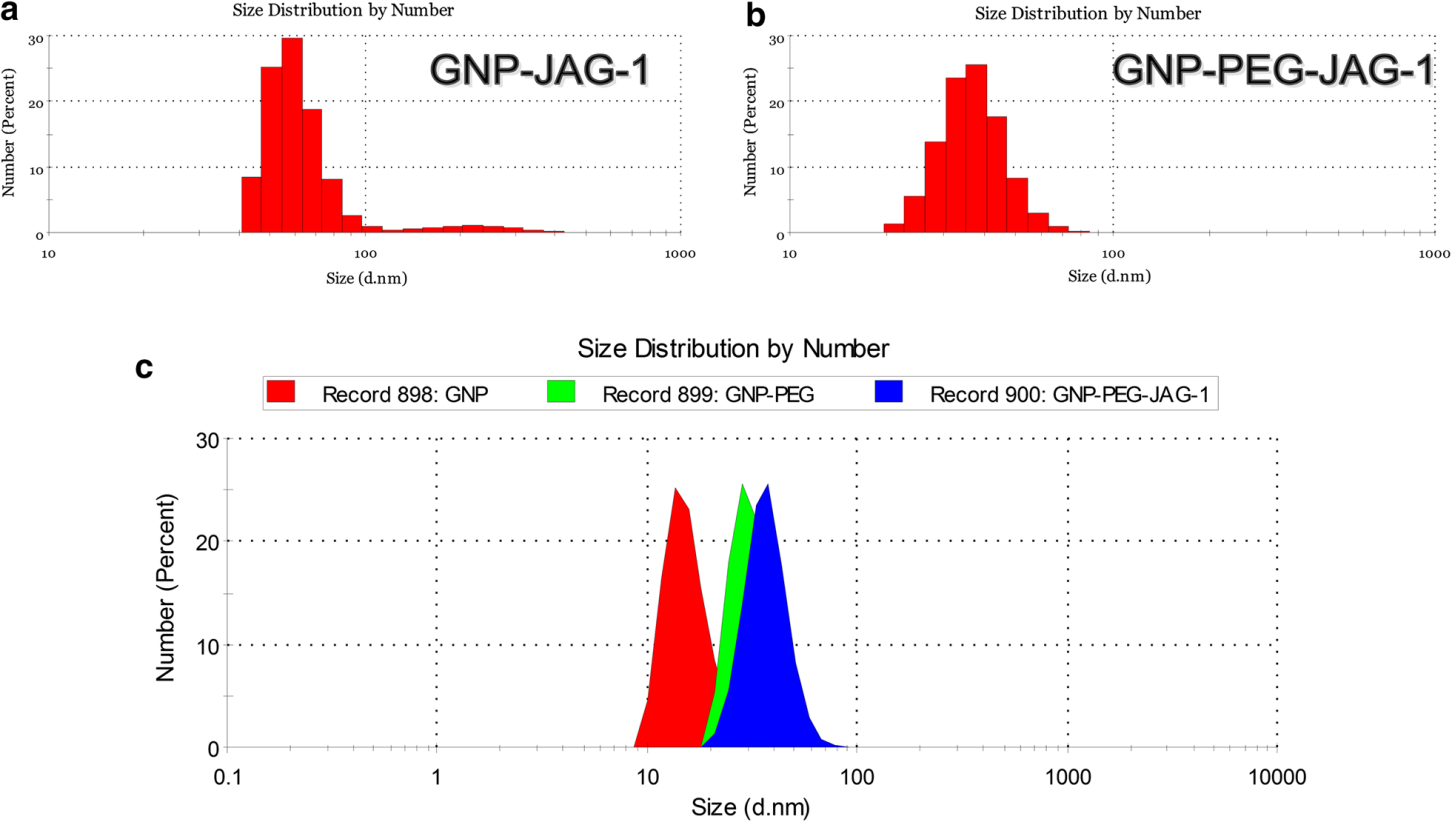
Dynamic light scattering (DLS) size distribution histogram of **a** GNP-JAG-1 sample; **b** GNP-PEG-JAG-1 sample. **c** Size distribution curves determined by DLS technique for citrate capped GNPs (*red*), PEG capped GNPs (*green*) and GNP-PEG-JAG-1 bio-nanocomposite (*blue*).

Dynamic light scattering (DLS) was used to determine the hydrodynamic diameter and polydispersity index of both prepared samples. Figure 3a presents a histogram for the GNP-JAG-1 sample as measured by DLS. Two nanoparticle populations are evident in this case, one having a 60 nm diameter (93.8%) and another population of JAG-1 functionalized nanoparticles with a diameter of 228 nm (6.2%). In the case of GNP-PEG-JAG-1, the DLS size distribution histogram shown in Figure 3b, a single monodisperse population with a hydrodynamic diameter of 37 ± 5 nm was registered. The size distribution curves for citrate capped GNPs, PEG capped GNPs and GNP-PEG-JAG-1 are presented in Figure 3c. The PEG capped GNPs increased in size, when compared with the citrate capped ones, having a 32 nm diameter.

**Figure 4:**
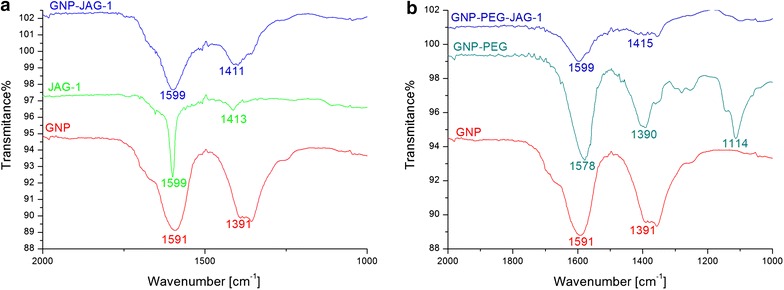
**a** ATR-FT-IR recorded spectra for citrate capped GNPs (*red line*), JAG-1 peptide (*green line*) and GNP-JAG-1 (*blue line*). **b** ATR-FT-IR recorded spectra for citrate capped GNPs (*red line*), PEG capped GNPs (*dark-green line*) and GNP-PEG-JAG-1 bio-nanocomposite (*blue line*).

The synthetized nanostructures were also investigated by ATR-FT-IR spectroscopy. Spectra for citrate capped GNPs, JAG-1 peptide and GNP-JAG-1 are given in Figure 4a. The IR spectra recorded for the GNP sample has two characteristic bands at 1,591 cm^−1^ and at 1,391 cm^−1^ corresponding to the antisymmetric and symmetric stretching vibrations of COO^−^ from the citrate ions present on the gold nanoparticle surface [31, 32]. The JAG-1 peptide presented an absorption band at 1,599 cm^−1^ which is attributed mainly to the C–C stretching vibrations from tyrosine [33] and phenylalanine [34] residues and a band at 1,413 cm^−1^ which is assigned to the C–N stretching vibrations from the proline [35] residue. The IR spectra of GNP-JAG-1 has a 1,599 cm^−1^ absorption band consistent with the one found for the JAG-1 peptide at the same wavenumber. As for the band registered at 1,413 cm^−1^ for JAG-1, in the case of JAG-1 coupled on the surface of GNPs the band has shifted to 1,411 cm^−1^. The comparison of GNP and GNP-JAG-1 IR spectra clearly indicates the presence of the JAG-1 peptide on the gold nanoparticle surface.

In the case of GNP-PEG-JAG-1 we first compared the spectra of citrate capped GNPs (Figure 4b—red line) versus PEG capped GNPs (Figure 4b—dark-green line). The initial 1,591 and 1,391 cm^−1^ absorption bands intensities, coming from the initial citrate layer of the GNPs, are shifted, after PEG dithiol addition, to 1,578 and 1,390 cm^−1^, respectively. This, and the appearance of an absorption band at 1,114 cm^−1^ which is attributed to the backbone C–O–C stretching of PEG [36] confirms the successful PEGylation of the gold nanoparticles. The JAG-1 coupling onto the GNP-PEG nanostructures is confirmed by the presence of the peptides ‘fingerprint’ absorption bands at 1,599 and 1,415 cm^−1^ in the GNP-PEG-JAG-1 sample.

**Figure 5:**
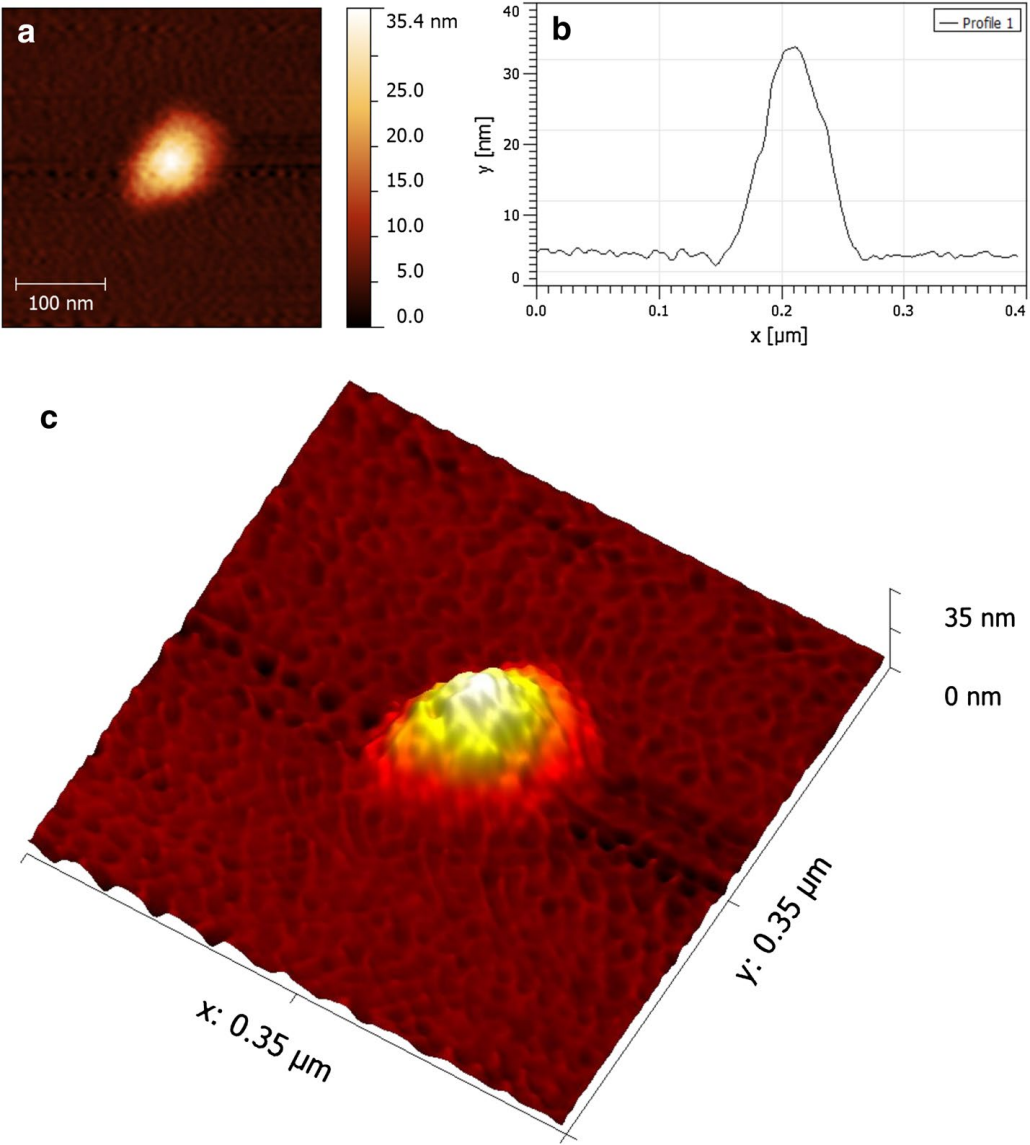
AFM measurement of GNP-PEG-JAG-1: **a** 2D image (*scale bar* 100 nm); **b** cross section graph of a single GNP-PEG-JAG-1 nanoparticle; **c** 3D rendering of GNP-PEG-JAG-1.

Atomic force microscopy (AFM) measurements were conducted on the monodisperse GNP-PEG-JAG-1 sample, in order to further understand the shape and size of the obtained bio-nanostructures. Figure 5a depicts a 2D representation of GNP-PEG-JAG-1, while Figure 5c depicts a 3D representation of the same bio-functionalized nanoparticle. In both cases, spherical shaped nanoparticles with an average diameter of 34 nm can be observed. The difference in size between the DLS and AFM measurements can be attributed to the fact that the DLS technique gives information about the hydrodynamic diameter of the gold core surrounded by the organic layer and the solvation layers [37]. The cross section graph of a representative GNP-PEG-JAG-1 nanoparticle is presented in Figure 5b. Also a comparison of the AFM images obtained for the GNP, GNP-JAG-1 and GNP-PEG-JAG-1 samples is presented in Additional file 1: Figure S1.

**Figure 6:**
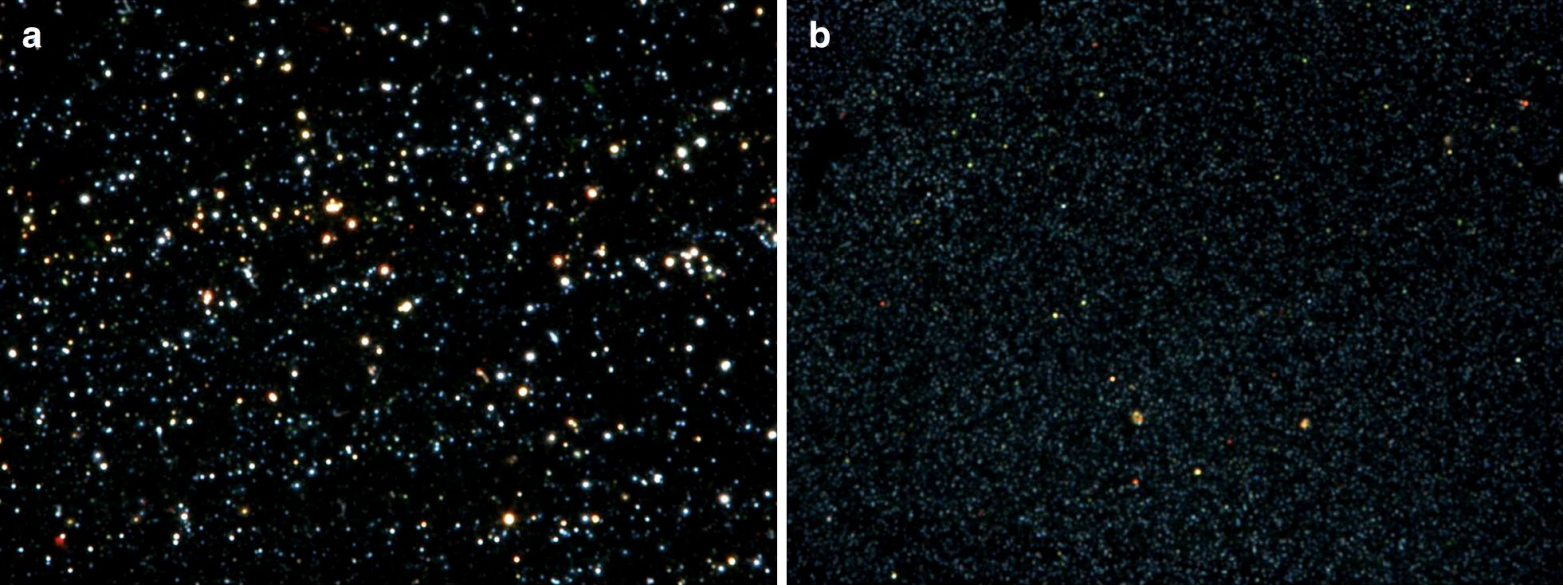
High contrast dark-field microscopy images for **a** citrate capped GNPs sample and **b** GNP-PEG-JAG-1 bio-nanocomposite.

High contrast dark-field microscopy was used din order to investigate the light scattering characteristics for citrate capped GNPs (Figure 6a) and for the GNP-PEG-JAG-1 bio-nanostructure (Figure 6b) in DI water. The latter one shows a scattering resonance red shift after the functionalization step, an indication of diameter size increase.

**Figure 7:**
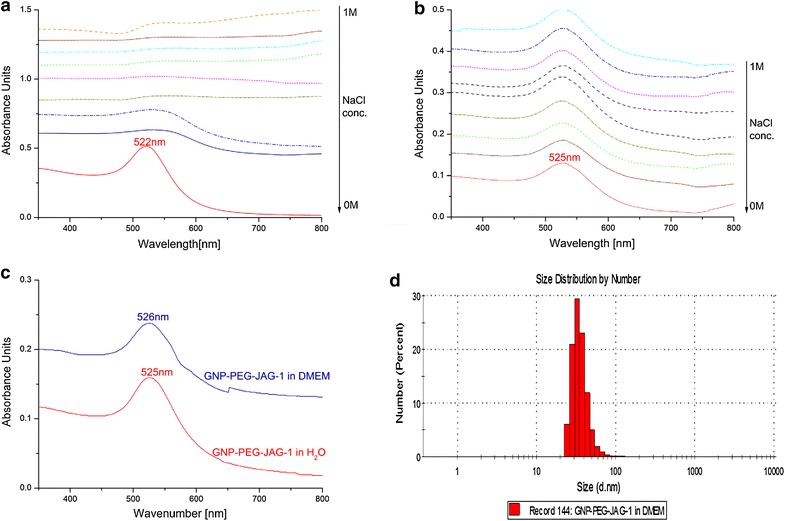
**a** UV–Vis spectra of citrate capped GNPs dispersed in NaCl solutions (concentrations ranging from 0 to 1,000 mM NaCl); **b** UV–Vis spectra of GNP-PEG-JAG-1 dispersed in NaCl solutions (concentrations ranging from 0 to 1,000 mM NaCl); **c** UV–Vis spectra of GNP-PEG-JAG-1 dispersed in water (*red line*) versus GNP-PEG-JAG-1 dispersed in DMEM (*blue line*); **d** DLS size size distribution histogram of GNP-PEG-JAG-1 dispersed in DMEM.

The hydrodynamic stability of the GNP and GNP-PEG-JAG-1 samples were evaluated in NaCl solutions with various concentrations (NaCl 0 mM; 9.625 mM; 19.25 mM; 38.5 mM; 77 mM; 154 mM; 308 mM; 500 mM; 1,000 mM) by means of UV–Vis spectroscopy. In Figure 7a The position of the SPR band for the GNP sample was affected by the presence of NaCl ions in the dispersing medium; at a 0 mM NaCl concentration the λ_max_ = 522 nm, this peak decreases in intensity inversely proportional to the NaCl concentration. This phenomena can be explained by the fact that the citrate capped GNPs tend to agglomerate in presence of NaCl. By contrast, the UV–Vis spectra of the GNP-PEG-JAG-1 sample (Figure 7b) exposed to various concentrations of NaCl solutions (NaCl 0 mM; 9.625 mM; 19.25 mM; 38.5 mM; 77 mM; 154 mM; 308 mM; 500 mM; 1,000 mM) remains unchanged regardless of the NaCl concentrations. The GNP-PEG-JAG-1 sample stability was also tested in DMEM culture media and monitored by means of UV–Vis spectroscopy and DLS techniques. Figure 7c depicts the UV–Vis spectra of GNP-PEG-JAG-1 in H_2_O (red line) and DMEM cell culture media (blue line), in both cases the SPR band was centered at around 525 nm indicating no agglomeration of the PEG-JAG-1 functionalized gold nanoparticles. This is in agreement with the DLS results, depicted in Figure 7d, GNP-PEG-JAG-1 in DMEM cell culture media registered a single monodisperse population with a hydrodynamic diameter of 36 ± 9 nm.

**Figure 8:**
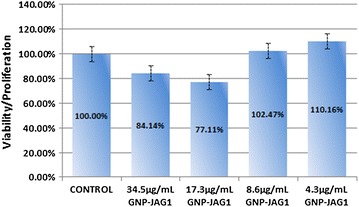
MTT viability/proliferation assay of MC3T3-E1 osteoblast cells exposed to different concentrations of GNP-PEG-JAG-1.

No significant change in viability/proliferation rate was observed for the MC3T3-E1 osteoblast cell line after exposure to various concentrations of nanomaterial (across group significance p = 0.591; control vs 34.5 μg mL^−1^ p = 0.077, control vs 4.3 μg mL^−1^ p = 0.376). However, a bimodal effect could be observed: while higher concentrations of the bio-nanostructure decreased the proliferation rate (34.5 μg mL^−1^ GNP-PEG-JAG-1 and 17.3 μg mL^−1^ GNP-PEG-JAG-1), the lower concentration values tend to promote cell proliferation (8.6 μg mL^−1^ GNP-PEG-JAG-1, 4.3 μg mL^−1^ GNP-PEG-JAG-1), as shown in Figure 8.

## Discussions

The objective of our present study was to develop a rational route to functionalize gold nanoparticles with the JAG-1 protein active peptide fragment. Two routes were proposed and investigated in terms of the synthetized bio-nanostructures.

Gold nanoparticles with different sizes and functionalities were developed and analyzed in recent years for a wide range of biomedical applications [7], one of them being the transport and unloading of pharmaceuticals. In a study conducted by Wang et al. GNPs were shown to greatly enhance intracellular peptide delivery [38].

The JAG-1 peptide is a Notch agonist with in vitro activity corresponding to JAGGED-1 protein residues 188–204 and is a part of the Delta/Serrate/LAG-2 region [2]. The 17 aminoacid JAG-1 peptide (Figure 1—top-side) has three cysteine residues in its structure. These thiol containing residues were used in order to couple the peptide on the gold nanoparticle surface. Even though the formation GNP-peptide conjugates, (via cysteine) has been previously described in literature [39, 40], more recent studies suggest the phenomena is not fully understood and the Au–sulphur interactions may be partially covalent and partially noncovalent [38, 41, 42].

Firstly, we have synthesized citrate capped GNPs because of their excellent size distribution very good stability in aqueous media and their ability to react with a broad range of ligands [27]. UV–Vis measurements for the synthesized GNPs showed a SPR band located at 522 nm, DLS measurements confirmed the monodisperse character and their 16 nm size, while the ATR-FT-IR data confirmed the presence of citrate ions on the surface of the gold nanoparticles.

In the first route, the JAG-1 peptide was directly coupled on the GNPs via thiol–Au interaction. The synthetized citrate capped GNPs were introduced to JAG-1 under vigorous agitation and neutral pH. The resulting GNP-JAG-1 sample had UV–Vis spectra with two SPR bands, one at 535 nm and another one at 652 nm. The second SPR band is attributed to the dipole coupling between plasmons of neighboring particles forming aggregates [43]. The formation of aggregates when JAG-1 was added to citrate capped GNPs is also confirmed by the DLS measurements who revealed a polydisperse GNP-JAG-1 sample with two populations centered at 60 and 228 nm. The addition of peptides to citrate-capped GNPs can lead to highly stable peptide-capped GNPs or, as in this case, in the formation of aggregates, depending on the peptide structure [43]. Although the GNP-JAG-1 bio-nanostructure proved polydisperse, ATR-FT-IR investigations were carried out in order to find out if indeed the JAG-1 peptide was coupled onto the GNPs. The IR bands located at 1,599 and 1,411 cm^−1^ confirmed this.

The second route employed for the JAG-1 functionalization of GNPs involved the place-exchange reaction between citrate ions present on the GNPs and dithiol PEG. The reaction was monitored via UV–Vis, ATR-FT-IR and DLS. The PEGylated GNPs presented themselves as monodisperse, with a hydrodynamic diameter of 32 nm and an IR absorption band at 1,114 cm^−1^ attributed to the backbone C–O–C stretching of PEG. The second step of this route was to bind cysteine terminated JAG-1 peptide with the PEGylated GNPs. In order to achieve this JAG-1 was introduced to GNP-PEG in the presence of DMSO as an oxidizing agent so that a disulfide bond was formed between the cysteine from JAG-1 and the thiol terminated PEG of the GNPs.

The UV–Vis spectra of GNP-PEG-JAG-1 presented a SPR band at 525 nm, this bathochromic shift indicates the place exchange reaction occurring between the PEG dithiol and the citrate ions initially found on the GNPs. DLS measurements for citrate capped GNPs, PEGylated GNPs and GNP-PEG-JAG-1 samples showed them as being monodisperse with hydrodynamic diameters of 16, 32 and 37 nm respectively. Confirmation of the PEGylation and JAG-1 functionalization steps also came from the ATR-FT-IR spectras, data presented in the results section. AFM measurements conducted on the GNP-PEG-JAG-1 sample showed the synthetized nanostructure as being spherical in shape.

The results obtained showed that the presence of a PEG spacer between the JAG-1 peptide and the gold core yields a monodisperse, aqueous stable nanometric structure. Moreover, biological activity results reveal no significant toxicity and low GNP-PEG-JAG-1 concentrations tend to induce the intended osteoblast—stimulatory effect. The lack of cytotoxic significance could be due to the limited 1-h incubation used. Further strategies should be tried to optimize this effect (exposure time prolongation, chronic exposure, etc.). Further research studies are needed to asses the Notch pathway activation potential of this novel bio-nanostructure. This study indicates a new strategy in delivering protein active peptide fragments with the aid of gold nanoparticles.

## Conclusions

We have investigated two functionalization routes to couple the JAG-1 peptide with gold nanoparticles. The hetero-functionalized GNP-PEG-JAG-1 bio-nanostructure obtained had a 37 nm diameter and was stable in aqueous media. Our results could have a significant impact in the assembly routes employed to bind biological active peptides to gold nanoparticles. The activation potential of the Notch pathway by this newly synthetized bio-nanocomposite is under investigation by our research group.

## Additional files

Additional file 1:
**Figure S1.** AFM images of citrate capped GNPs (left image), JAG-1 functionalized GNPs (centre image) and PEG-JAG-1 functionalized GNPs (right image).
