# Supplementary material for: Rational design of gold nanocarrier for the delivery of JAG-1 peptide
Source: J Nanobiotechnology. 2015 Jun 16;13:41. doi: 10.1186/s12951-015-0100-x (PMC4469399; doi:10.1186/s12951-015-0100-x)
Supplement: Additional file 1: — Figure S1. AFM images of citrate capped GNPs (left image), JAG-1 functionalized GNPs (centre image) and PEG-JAG-1 functionalized GNPs (right image). [file 12951_2015_100_MOESM1_ESM.docx]

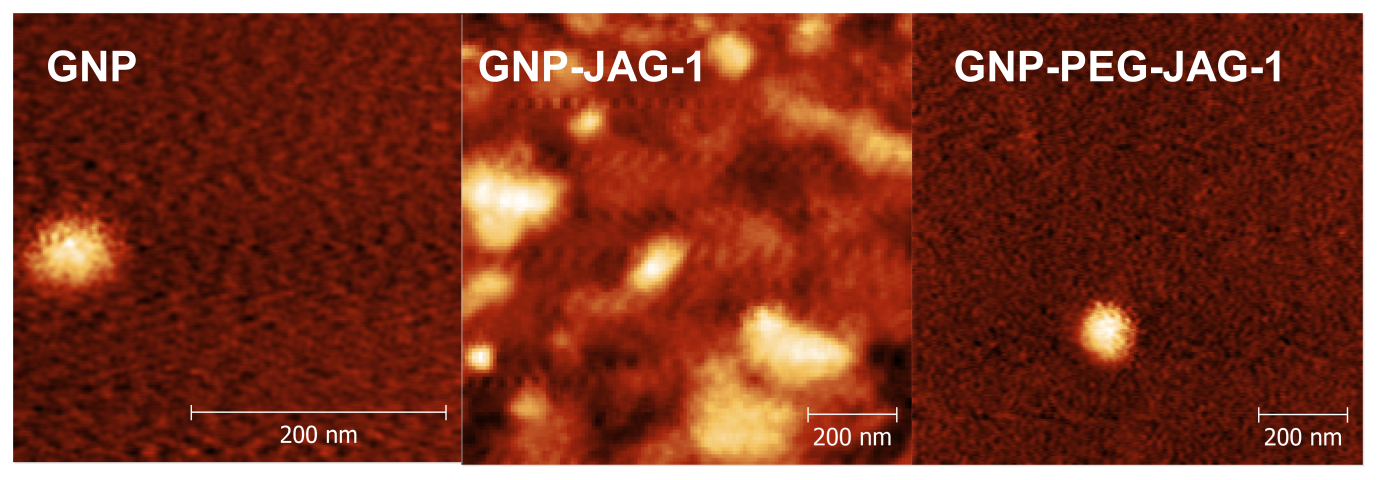


**Figure S1.** AFM images of citrate capped GNPs (left image), JAG-1 functionalized GNPs (centre image) and PEG-JAG-1 functionalized GNPs (right image).

In figure S1. a citrate caped gold nanoparticle (GNP) is shown in the left image, the size and shape registered are in agreement with data provided by the DLS and UV-Vis techniques. The GNP-JAG-1 (figure S1- centre image) shows that a direct functionalization approach of JAG-1 onto the GNP surface yields irregular nanostructures with sizes ranging from around 60nm to 220nm. This is also in agreement with the data obtained for GNP-JAG-1 by UV-Vis spectroscopy and DLS techniques.
